# PERK Pathway and Neurodegenerative Disease: To Inhibit or to Activate?

**DOI:** 10.3390/biom11030354

**Published:** 2021-02-26

**Authors:** Talya Shacham, Chaitanya Patel, Gerardo Z. Lederkremer

**Affiliations:** 1 Cell Biology Division, George Wise Faculty of Life Sciences, The Shmunis School of Biomedicine and Cancer Research, Tel Aviv University, Tel Aviv 69978, Israel; talyash1@mail.tau.ac.il (T.S.); chaitanyspatel@gmail.com (C.P.); 2 Sagol School of Neuroscience, Tel Aviv University, Tel Aviv 69978, Israel

**Keywords:** ER stress, unfolded protein response, integrated stress response, eIF2, Alzheimer’s disease, Parkinson’s disease, Huntington’s disease, ALS

## Abstract

With the extension of life span in recent decades, there is an increasing burden of late-onset neurodegenerative diseases, for which effective treatments are lacking. Neurodegenerative diseases include the widespread Alzheimer’s disease (AD) and Parkinson’s disease (PD), the less frequent Huntington’s disease (HD) and Amyotrophic Lateral Sclerosis (ALS) and also rare early-onset diseases linked to mutations that cause protein aggregation or loss of function in genes that maintain protein homeostasis. The difficulties in applying gene therapy approaches to tackle these diseases is drawing increasing attention to strategies that aim to inhibit cellular toxicity and restore homeostasis by intervening in cellular pathways. These include the unfolded protein response (UPR), activated in response to endoplasmic reticulum (ER) stress, a cellular affliction that is shared by these diseases. Special focus is turned to the PKR-like ER kinase (PERK) pathway of the UPR as a target for intervention. However, the complexity of the pathway and its ability to promote cell survival or death, depending on ER stress resolution, has led to some confusion in conflicting studies. Both inhibition and activation of the PERK pathway have been reported to be beneficial in disease models, although there are also some reports where they are counterproductive. Although with the current knowledge a definitive answer cannot be given on whether it is better to activate or to inhibit the pathway, the most encouraging strategies appear to rely on boosting some steps without compromising downstream recovery.

## 1. Introduction

The accumulation of unfolded or misfolded secretory proteins, which causes a serious disturbance in endoplasmic reticulum (ER) function, termed ER stress, is a common feature in neurodegenerative diseases [1,2,3]. ER stress activates the UPR, which we will detail later on. The unfolded protein response (UPR) initially triggers cell-protective cascades, aimed at reducing the ER load of unfolded proteins, by transiently inhibiting protein synthesis and upregulating the protein folding and degradation machineries. Because the cell insult remains, usually in the form of mutant misfolded aggregation-prone proteins, the ER stress is not resolved, and the prolonged UPR initiates in the long-term pro-apoptotic processes, leading to cell death. Owing to insufficient compensatory mechanisms and scarce regeneration, the consequences in the central nervous system are profound, and are a main cause of neurodegeneration.

Ongoing gene therapy approaches, especially in the case of monogenic diseases such as Huntington’s disease (HD), attempt to reduce or eliminate the pathogenic mutant proteins, using siRNA [4], antisense knockdown [5] or allele-specific CRISPR/Cas9-mediated gene editing [6]. However, there is still a roadblock in attaining efficient delivery. Another approach centers on blocking the cellular toxicity caused by the misfolded proteins. Reduction of ER stress is an attractive aim, and can be accomplished by several strategies. One involves the targeting of the UPR pathways.

ER stress in neurodegenerative diseases activates all three pathways of the mammalian UPR, which we will detail below. However, it is becoming apparent that the PERK pathway has a main role in the generation and also in the resolution of the ensuing cytotoxicity. The PERK pathway is an increasing target for many studies that try to develop therapeutic approaches for neurodegenerative diseases that have so far remained refractory to any effective treatment. These include major, widespread diseases, such as AD, PD and ALS, as well as more circumscribed diseases such as HD and rare genetic diseases such as vanishing white matter disease (VWMD) and spinocerebellar ataxias. Perplexingly, multiple reports in recent years have successfully applied approaches that either inhibit or activate the PERK pathway in a variety of diseases or conditions. This review will focus on the dichotomies involved, i.e., the advantages and disadvantages in these approaches.

## 2. The Unfolded Protein Response

Protein misfolding, originated in gene mutations or in prion transmission, viral infection, DNA damage, reactive oxygen species (ROS) and other environmental and physiological factors, is responsible for induction of the cellular stress responses [7,8]. Proper protein folding, processing, localization and degradation are all crucial in maintaining protein homeostasis (proteostasis) within a cell. Disruption of proteostasis results in the activation of the cellular stress responses [9]. Accumulation of misfolded proteins in the ER results in ER stress and induction of the UPR, whereas the accumulation of misfolded proteins in the cytosol induces the Heat Shock Response (HSR) [10]. These stress responses are responsible for reducing the unfolded protein load by either halting protein synthesis, by increasing the expression of molecular chaperones to increase folding capacity or by upregulating the protein degradation machinery. The extent of ER stress and the ability to compensate it results in a selective role of the UPR, either pro-adaptive or pro-apoptotic. In case of failure in bringing the cell to homeostasis by the above methods, the UPR triggers initiation of programmed cell death or apoptosis [11,12].

**Figure 1:**
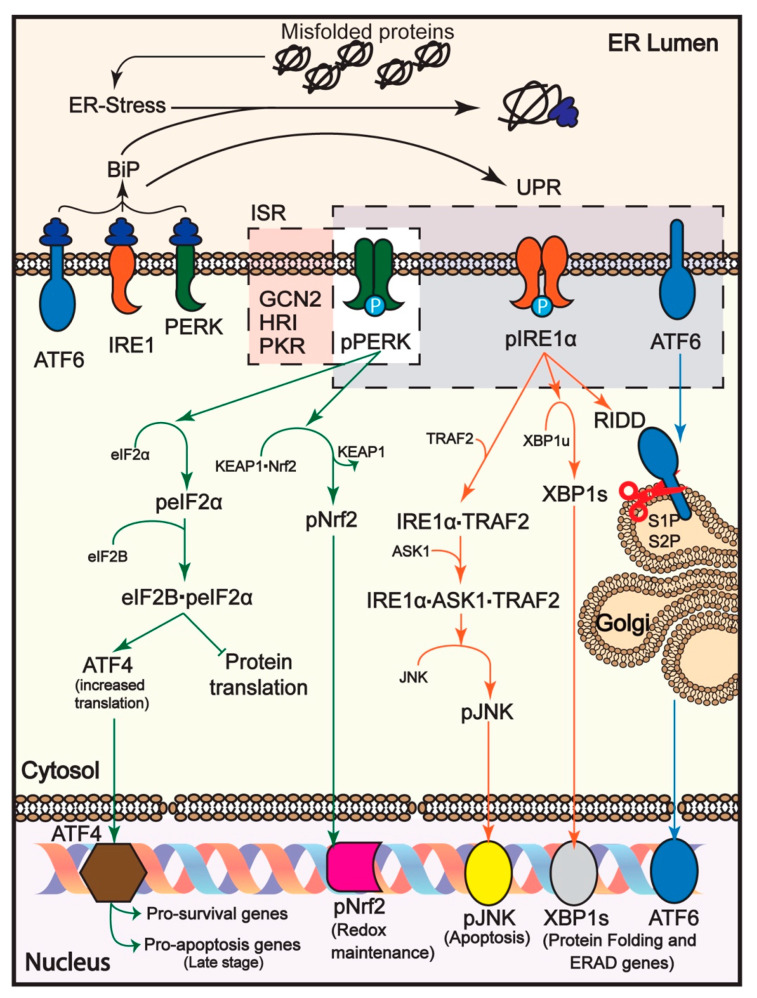
Unfolded protein response (UPR) pathways. Accumulated misfolded proteins in the ER cause ER stress, binding BiP and activating the UPR sensors, PERK, IRE1 and ATF6. Activated PERK initiates in the short-term a pro-survival outcome by inhibiting protein translation (reducing ER protein load), activating ATF4-dependent transcription of pro-survival genes and a Nrf2-dependendent redox maintenance pathway. In the long term, if ER stress persists, there is activation of pro-apoptotic genes. Activated IRE1 (exemplified here by the prevalent form IRE1α) also induces pro-survival genes to increase protein folding and ERAD capacity. This is achieved through IRE1-dependent splicing of unspliced XBP1 mRNA (XBP1u) to a spliced form (XBP1s), which encodes an active transcription factor. Regulated IRE1 Dependent Decay (RIDD) degrades mRNAs encoding for secretory proteins, reducing the ER load. In this case, there is also activation of pro-apoptotic genes, if ER stress persists, via Jun-N-terminal kinase (JNK) phosphorylation and activation by a complex of IRE1α with TRAF2 and apoptotic-signaling kinase-1 (ASK1). Activated ATF6 is transported to the Golgi complex for cleavage by site 1 and site 2 proteases (S1P, S2P) resulting in an active form released from the membrane, which traffics to the nucleus, inducing protein folding and ERAD genes.

The mammalian UPR is branched into three pathways, each with its UPR sensor, PERK [13], inositol-requiring transmembrane kinase/endoribonuclease 1 (IRE1) [14] and activating transcription factor 6 (ATF6) [15] (Figure 1). These three transmembrane proteins are in an inactive state when bound with the ER chaperone BiP (GRP78, 78 KDa glucose-regulated protein), under normal cellular conditions. Upon binding of a misfolded protein to BiP or directly to the UPR sensor, PERK, IRE1 and ATF6 are released and, thus, activated by dimerization and autophosphorylation for PERK and IRE1, and intermembrane proteolysis for ATF6 [12,16].

The activation of PERK results in the phosphorylation of the α subunit of eukaryotic translation initiation factor (eIF2α) rendering P-eIF2α (also referred to as eIF2(α-P)), which in turn transiently halts the synthesis of most cellular proteins by binding to the eIF2B guanine nucleotide exchange factor [16,17]. The P-eIF2α-eIF2B complex inhibits the binding of eIF2 to the initiator Met-tRNA, therefore reducing the ternary complex (eIF2-GTP-Met-tRNA) and inhibiting protein synthesis [18]. Nevertheless, the synthesis of a limited number of proteins is increased, among them the transcription factor 4 (ATF4), C/EBP Homologous protein (CHOP) and growth arrest and DNA damage-inducible protein 34 kDa (GADD34, also called PPP1R15A). The increase in the translation of ATF4 and others is due to the presence of upstream open reading frames (uORFs) in their 5′UTR [19]. Depending on the dynamics of PERK pathway activation, it can have a pro-adaptive or pro-apoptotic role. Under a pro-adaptive role, the transient translation inhibition reduces ER load. ATF4 then acts through a negative feedback loop by inducing the expression of several genes, one of them the transcription factor CHOP, which in turn induces, among other genes, GADD34. GADD34 forms a complex with protein phosphatase 1 (PP1) resulting in the dephosphorylation of P-eIF2α, release of eIF2B, and thus, reactivation of cellular protein synthesis [20]. P-eIF2α can also be dephosphorylated by a complex of PP1 with a constitutive regulator, CReP (PPP1R15B) [21]. If during this cycle ER stress is reduced, proteostasis is restored. Under a pro-apoptotic role, when ER stress is not resolved, ATF4 increases the expression of CHOP to a level that results in the initiation of apoptosis. CHOP is phosphorylated by p38 MAPK, which promotes its role in apoptosis [22]. Another protein that was recently reported to follow the same uORF-dependent translation is QRICH1, the increased translation of which promotes apoptosis [23].

The PERK branch of the UPR is part of the conserved intracellular signaling network called the Integrated Stress Response (ISR) [16]. The ISR is induced by proteostasis defects, nutrient deprivation, viral infection and oxidative stress within the cell. The ISR acts through four eIF2α kinases, activated by different cellular stresses: PERK, PKR, HRI and GCN2 [16,24,25].

Other than its involvement in regulating translation, PERK also acts against oxidative stress. The transcription factor NF-E2-related factor 2 (Nrf2) is another substrate which is phosphorylated and activated by PERK. In normal conditions, Nrf2 is kept inactive by binding to an adaptor of Cullin 3-based E3 ubiquitin ligase, Kelch-like ECH-associated protein 1 (KEAP1). This complex is kept in the cytosol, and Nrf2 is targeted for ubiquitin-mediated degradation [26]. Upon Nrf2 phosphorylation, pNrf2 is released from KEAP1 and traffics to the nucleus, where it activates transcription of genes involved in detoxification, anti-oxidation and metabolism [27,28].

IRE1, the sensor of the second UPR branch, is activated by autophosphorylation and oligomerization upon ER stress. There are two IRE1 variants, IRE1α and IRE1β. IRE1α is the best-studied form, IRE1β being tissue-specific, expressed mostly in the digestive tract [29]. IRE1 activation enables its special endoribonuclease activity, responsible for splicing the mRNA that encodes transcription factor XBP1 [16,30]. XBP1s (spliced form) codes for an active transcription factor, which upregulates genes involved in protein folding and ERAD (e.g., HRD1) [31,32,33]. In the long term, the RNase activity of IRE1α becomes less specific and can degrade many mRNAs localized to the ER through a process termed Regulated IRE1 Dependent Decay (RIDD). RIDD can also reduce the stability of miRNAs and rRNAs [34,35]. Prolonged stress results in the activation of ASK1 and JNK by pIRE1α, promoting apoptosis.

The third UPR sensor, ATF6, is translocated to the Golgi compartment upon ER stress, where it is cleaved to an active transcription factor by the enzymes site 1 protease (S1P) and site 2 protease (S2P). The activated ATF6 induces the upregulation of ER chaperones and ERAD genes (e.g., BiP, HRD1, SEL1L, Herp) [31,32,36].

## 3. ER Stress in Neurodegenerative Diseases

Accumulated misfolded proteins in the ER are directed to the ER-associated degradation pathway (ERAD), which involves retrotranslocation to the cytosol, for ubiquitylation and proteasome-dependent degradation. Remarkably, although in most neurodegenerative diseases the mutant misfolded proteins are expressed in the cytosol and not in the ER, they indirectly cause ER stress, frequently by inhibiting ERAD [3]. The insufficiency of the UPR in compensating the ER stress leads in the long term to cell death.

Unlike most mammalian cell types, neurons have a very limited regeneration rate. Therefore, depletion of neurons due to cell death results in neurodegeneration, a loss of neuronal function in central nervous system tissues. Some of the best known examples of neurodegenerative diseases are AD, PD, HD, ALS, Prion Disease and tauopathies such as Progressive Supranuclear Palsy (PSP) and Frontotemporal Dementia (FTD) [11].

In AD, which is the most prevalent neurodegenerative disorder, pathogenesis is a result of environmental and genetic factors [37]. Manifestation of AD starts with memory impairment, which is caused by depletion of neurons in the hippocampal formation and para-hippocampal gyrus regions of the brain. The specific cell types most affected in AD pathology are the ones which interconnect the hippocampal formation with the association cortices, basal forebrain, thalamus and hypothalamus [38]. AD has been linked to hyperphosphorylation of the Tau protein (pTau), which destabilizes neuronal microtubules causing intracellular neurofibrillary tangles (NFT), and extracellular plaques caused by accumulation of Amyloid-β (Aβ) peptides, due to mutations in the amyloid precursor protein (APP) [37,39,40]. Toxic soluble pTau, Aβ-oligomers and NFT were shown to inhibit ERAD and the proteasome machinery [41,42]. This results in the increase in protein load within the ER, inducing ER stress and activating the UPR [43,44,45] (Table 1). BiP, pPERK, pIRE1α, P-eIF2α, ATF4 and beta-site APP cleaving enzyme 1 (BACE1) have been found to be upregulated in AD models. Prolonged PERK branch activation in AD was shown to affect memory and promote neurodegeneration by affecting protein synthesis [46,47]. It is still unclear why specific brain regions and cell types are more affected [48]. ER and oxidative stress induced by Aβ/pTau can also lead to activation of the ASK1 branch of the IRE1α pathway [49,50]. Under prolonged ER stress, AD brains showed upregulation of pro-apoptotic pathways, especially increased CHOP expression, causing induction of oxidative stress, which further resulted in an increase of Aβ-oligomers and neuronal death [51]. A recent study reported that in the brains of Down Syndrome patients (who have a high propensity to develop AD), there is sustained activation of the PERK pathway, but it fails to regulate anti-oxidant outcomes through Nrf2, therefore exacerbating oxidative stress [52].

PD can be caused by mutations in the SNCA gene, which codes for α-synuclein, resulting in the accumulation of mutant α-synuclein in so-called Lewy bodies in the dopaminergic (DA) neurons in the substantia nigra pars compacta (SNpc), causing neuronal death [44,53]. The activation of the PERK pathway through increased levels of pPERK and P-eIF2α in patient brains carrying PD and in cellular models has been reported by several studies, suggesting a pro-apoptotic role of the PERK branch in PD [53,54,55]. Although α-synuclein is not an ER resident protein, it has been reported to interact directly with the machineries involved in vesicular transport, with the ER/Golgi membranes [56] and the outer mitochondrial membrane [69]. α-Synuclein has been shown to activate the UPR through several mechanisms: (1) Some studies showed that α-synuclein oligomers are responsible for the inhibition of the proteasome machinery, (2) α-synuclein aggregates were reported to interact directly with BiP and activate the UPR in PD, although it is unclear how they are translocated into the ER [56] and (3) α-synuclein interacts with RAB1 [70], impairing COPII vesicular trafficking, and therefore, inhibiting ATF6 activation and blocking this pro-adaptive branch of the UPR, leading to apoptosis [57]. Therefore, a simultaneous targeting of the PERK and ATF6 UPR branches could be a possible therapeutic strategy for treating PD.

In models of the rare neurodegenerative disease VWMD, caused by mutations in eIF2B, there is a pernicious downstream effect on the ISR, leading to demyelination of neurons in the white matter of the CNS [71]. Reduction of activity in mutant eIF2B has a similar effect as sustained eIF2α phosphorylation, inhibiting protein synthesis and causing activation of the ATF4 pro-apoptotic outcomes [58,59].

HD is a neurodegenerative disorder caused by the aggregation of mutant huntingtin (mHtt), resulting in a selective neuronal death, starting in the striatum but also extending to the cortex and some other areas of the brain [72,73,74]. mHtt was shown to cause ER stress and upregulation of UPR markers such as pPERK, P-eIF2α, CHOP, GADD34, BiP, ATF6 and XBP1s in HD models [75,76]. ER stress was observed in HD cellular models [60,61,77,78,79], in HD animal models [77,80,81,82], and in postmortem samples from HD patients [77], reviewed in [75,76,83,84]. As mHtt is present in the cytosol and nucleus and not in the ER, the question arises of how it causes ER stress. mHtt was shown to sequester and deplete the cytosolic chaperone p97/VCP and its cofactors Npl4 and Ufd1, which are essential for ERAD. ERAD inhibition leads in turn to protein accumulation in the ER and ER stress [61,78,85]. A similar mechanism of p97 sequestration was observed in a model of polyQ expanded Spinocerebellar ataxia type-3 (SCA3) disease [86]. In the case of HD, soluble mHtt oligomers were found to be the causative agent of ER stress [61] and the main UPR pathway induced was the PERK pathway [60]. As in other neurodegenerative diseases, it is still unclear why specific cell types are more vulnerable, but a very low activity of PERK-mediated eIF2α phosphorylation in striatal neurons was connected to the higher mHtt toxicity in this region [60]. The observed increase in eIF2α phosphorylation in the presence of mHtt was thought to be detrimental, but it was later concluded that it is actually an insufficient cellular attempt to restore homeostasis [87]. Other UPR pathways are also involved; when the IRE1 pathway was compromised, there was compensation by an increase in autophagy, which would help to clear misfolded mHtt [82].

ALS is a fatal neurodegenerative disorder which affects large motor neurons of the brain and spinal cord. Although it is mainly a sporadic disease, about 10% of ALS cases are familial in nature. Familial ALS can be caused by mutation in several genes, including chromosome 9 open reading frame 72 (C9ORF72) [65], TAR DNA binding protein 43 (TDP-43) [67], the RNA binding protein fused in sarcoma (FUS) [66], superoxide dismutase-1 (SOD1) [88] and Ubiquilin-2 (UBQLN2) [44,89,90]. UBQLN2 has a role in targeting misfolded proteins in the cytosol and the nucleus to proteasomal degradation [90]. In addition to ALS, mutations in UBQLN2 have also been associated with FTD. It is involved in the formation of stress granules [91]. Mutant FUS and TDP43 also accumulate in the cytosol in the form of stress granules and induce ER stress [67]. Mutant SOD1 (mSOD1) leads to ALS pathogenicity by causing ER stress and especially by activating the PERK pathway through several mechanisms [92]: (1) mSOD1 interferes with COPII vesicular transport [93], (2) mSOD1 showed interaction with Derlin1, an ER membrane protein involved in ERAD, which impaired the ERAD pathway in ALS models [63,64,94], (3) accumulation of mutant SOD1 was reported in the ER lumen, where it binds BiP, inducing ER-stress. In the latter mechanism, a problem of topology arises, similar to that mentioned above with α-synuclein, as it is unclear how cytosolic SOD1 is translocated into the ER lumen. It was reported that PERK haploinsufficiency has a deleterious effect on mSOD1 model mice [95], but these results were challenged in a recent study, which showed no significant effects in disease progression [96].

Prion diseases, also known as transmissible spongiform encephalopathies (TSEs), are characterized by lesions with spongiform changes, gliosis and neuronal loss [97]. It is caused by the development of a protease resistant form of an abnormally folded cellular prion protein (PrP), leading to its aggregation and induction of ER stress [44,98,99]. All the branches of the UPR were shown upregulated in prion disease models, especially the PERK/eIF2α pathway. In cellular models, upregulation of XBP1s and ATF6 showed protective effect from PrP aggregates. GADD34 overexpression was protective, suggesting that prolonged eIF2α phosphorylation is an important factor in prion pathogenicity [68].

The delicate balance of the cell environment is critical when considering the UPR machinery, and especially the PERK pathway, as therapeutic targets in neurodegenerative diseases. While insufficient activation, as a physiological response to the disease, causes accumulation of unfolded proteins, which interfere with ER function, chronic activation in disease inhibits synthesis of new proteins, leading to their depletion, and activates pro-apoptotic pathways. Both conditions may lead to cell loss, with irreversible damage and neurodegeneration in the long term. Therefore, both approaches of activation or inhibition of the PERK pathway have been considered as potential therapies for a variety of diseases (reviewed in [2,16,30,100,101,102,103,104]).

## 4. PERK Pathway Activation

Activation of the PERK pathway results in transient protein synthesis inhibition, reducing ER protein load, and inducing cell protective pathways through ATF4 and Nrf2. Chronic reduced PERK activity is detrimental, as seen from PERK mutations in Wolcott-Rallison syndrome, which causes early-onset diabetes, epiphyseal dysplasias and neurodegeneration [105,106,107]. Additionally, in several tauopathies, PERK variants with reduced activity are a genetic risk factor with high vulnerability to ER stress in cells expressing them [108]. Therefore, targeted PERK pathway activation has been considered as a possible therapeutic approach.

**Figure 2:**
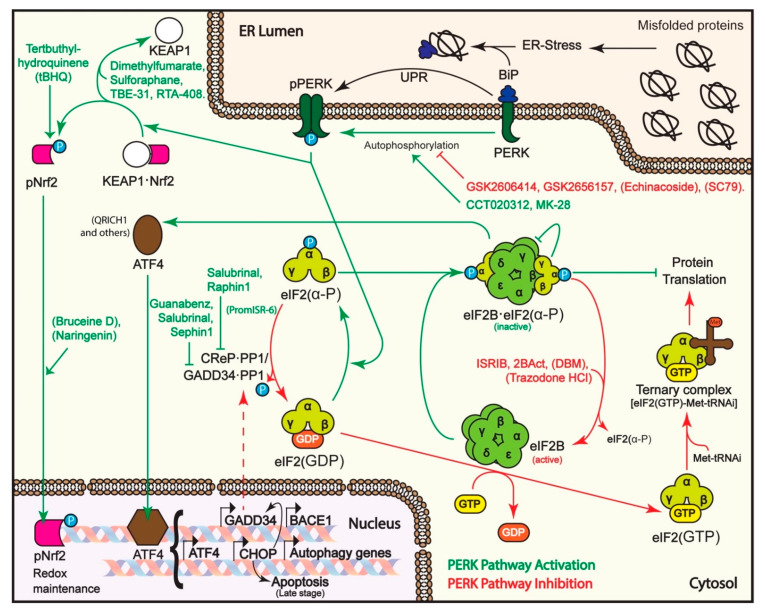
PERK pathway and small molecule modulators. UPR induction results in dimerization and autophosphorylation of PERK. pPERK catalyzes phosphorylation of the α subunit of the translation factor eIF2 (eIF2(α-P)), which associates with the guanine nucleotide exchange factor eIF2B, inhibiting its activity and, thus, causing an arrest in transient protein translation. However, this also leads to activation of the translation of ATF4 and other mRNAs with upstream ORFs. ATF4 induces transcription of pro-survival genes and also in the longer term of CHOP, a transcription factor that induces among others the transcription of GADD34. GADD34 forms a complex with PP1, leading to the dephosphorylation of eIF2(α-P) and resumption of protein synthesis. The constitutively expressed CReP also associates with PP1 to maintain a basal level of eIF2(α-P) dephosphorylation activity. Activated PERK also phosphorylates Nrf2, causing its release from KEAP1 and allowing its traffic to the nucleus, where pNrf2 induces transcription of redox maintenance genes. In the long term, if ER stress persists, CHOP and QRICH1 accumulation leads to the induction of pro-apoptotic genes. Arrows and modulators in green indicate steps and activators of the PERK pathway, while those in red show steps and compounds that turn off the pathway. Compounds with unknown target mechanism are shown in parentheses.

A first strategy that was tried for specific PERK pathway activation was the inhibition of GADD34. GADD34 deletion or expression of a dysfunctional GADD34 had shown beneficial effects in models of Charcot-Marie Tooth and familial ALS diseases [109,110]. GADD34 inhibition impedes the dephosphorylation of P-eIF2α, prolonging the arrest in protein synthesis. The first inhibitor that was identified was the small molecule salubrinal, which showed protection from ER stress in cellular and animal HD, PD, traumatic brain injury (TBI) and excitotoxic neuronal injury models [53,79,111,112,113,114] (Figure 2). Salubrinal also targets the constitutive PP1 regulatory subunit CReP [111] (Table 2). Guanabenz, a hypotensive drug acting on the β2 adrenergic receptor, showed enhanced effects [115] and was beneficial in familial ALS [116], VWMD [117] and in PD cellular and animal models, by increasing ATF4 levels, leading to upregulation of parkin [118,119,120]. GADD34 inhibition has also been tried in non-neurodegenerative diseases. For example, in cancer models, Salubrinal combined with 4E1RCat (a dual inhibitor of eIF4E:4E-BP1 and eIF4E:eIF4G) decreased protein synthesis in melanoma cells and impeded tumor growth in mice [121]. Guanabenz improved insulin resistance by upregulating hepatic LepRb expression (involved in lipogenesis and fatty acid β-oxidation) in models of nonalcoholic fatty liver disease [122]. An analogue of guanabenz, Sephin1, developed to remove the β2 adrenergic activity, also showed protective effects in Charcot-Marie-Tooth disease and in a model of familial ALS [123]. Sephin1 delayed the onset of clinical symptoms in a multiple sclerosis (MS) mouse model by inducing prolonged ISR [124], and extended survival of prion infected mice [125]. However, the target specificity of guanabenz and Sephin1 was later challenged [126]. PromISR-6 is another molecule recently found in an in silico screen of guanabenz analogues. Although its target was not identified, it prolonged eIF2α phosphorylation and protein translation inhibition, reducing mutant Htt aggregates and increasing survival in an HD cellular model, apparently by activating autophagy [127]. Raphin1, a drug developed to specifically target CReP, also showed protective effects in an HD mouse model [128].

The downside of inhibiting P-eIF2α phosphatases is that, as we have seen above, prolonged eIF2α phosphorylation causes extended inhibition of protein synthesis, with the consequent depletion of essential short-lived proteins and extended ATF4 upregulation, leading to expression of downstream pro-apoptotic factors. ATF4 overexpression causes cytotoxicity, as was seen by nigra-striatal degradation in PD animal models [181,182]. Conversely, ATF4- deficient dopaminergic neurons showed attenuated death under a PD neurotoxin and ATF4 inhibition reduced the production of proinflammatory cytokines by mouse microglia in culture [183]. In an MS mouse model, in experimental autoimmune encephalomyelitis, upon deletion of PERK, there was axon degeneration and loss. However, ATF4 inactivation did not show the same result, implying involvement of additional protective factors activated by PERK other than ATF4 [184].

As mentioned above, an additional PERK substrate, besides eIF2α, is Nrf2. Nrf2 activation has beneficial effects in neurodegenerative disease (reviewed in [185]). Several Nrf2 activators have shown protective effects, such as tertbuthyl-hydroquinene, which was shown to be beneficial in cellular models of PD [173], in a rat model of HD [171] and in AβPP/PS1 AD model transgenic mice [172]. Indirect Nrf2 activation was also achieved by sulforaphane (SFN), dimethylfumarate (DFM) and TBE-31. These drugs modify cysteine 151 in KEAP1, resulting in the release of Nrf2 [186]. TBE-31 showed protection by reducing oxidative stress in cellular models of Friedrich Ataxia (FRDA), caused by GAA repeat expansion leading to reduced levels of the mitochondrial protein frataxin [168].

SFN has shown neuroprotection in several disease models of PD [159,160,161], HD [162], AD [163,164,165,166], MS [167] and FRDA [168]. In cancer treatment, it protects against the side effects of chemotherapy by doxorubicin on the heart [187]. DFM is currently used as an oral therapeutic agent for the treatment of relapsing forms of MS [175]. DFM also showed protection in a PD mouse model [176,177]. RTA-408 (omaveloxolone) is currently in clinical trials for the treatment of FRDA [174]. Other drugs that indirectly induce Nrf2 signaling also showed neuroprotective effects; for example, Bruceine D, a drug used in cancer treatment [188,189], which was reported to cause significant improvement in motor function and reduced dopaminergic neuron loss in PD model mice [153]. Another drug that induces Nrf2 signaling, Naringenin, enhanced the neurotrophic effect of astroglia over dopaminergic neurons [156] and improved learning and memory in a rat AD model [157]. However, it was found to have additional targets [158].

Direct activation of PERK can boost the protective phases of the pathway without compromising long-term recovery of eIF2α function by dephosphorylation, as GADD34 is still induced. Direct activators of PERK were identified only recently. The compound CCT020312 was found in a phenotypic screen that assayed for G1/S checkpoint activators in human colon carcinoma cells [190]. It also inhibited triple-negative breast cancer by G1 phase cell cycle arrest [191]. CCT020312 showed neuroprotection in cellular and mouse models of tauopathies, reducing tau phosphorylation in P301S tau mice and significantly improving their memory and motor function, with a reduction in motoneuron loss [129]. Another compound, MK-28, was recently identified as a potent and selective PERK activator [87] in an evaluation of derivatives of a mother compound, A4, which had been found in an in vitro screen for PERK modulators [192]. MK-28 was the derivative that showed the strongest reduction in ER stress-induced apoptosis in a striatal cell line (ST*Hdh*^Q111/111^) [193] derived from knock-in HD model mice. It also showed much higher efficacy in vitro compared to CCT020312. In vivo MK-28 treatment reduced disease progression, significantly improving motor functions and increasing life expectancy in R6/2 HD model mice [87].

## 5. PERK Pathway Inhibition

We have described above the benefits of targeted PERK pathway activation using small molecule compounds, as it plays an important role in the ability of the cell to restore homeostasis upon ER stress. However, this is not the case in all studies. Although GADD34 inhibition (leading to PERK pathway activation) has proven beneficial in many studies, involving several neurodegenerative diseases, it was found to be detrimental in other reports. A study in prion-infected tg37 mice showed a negative effect of the GADD34 inhibitor salubrinal, with increased neurotoxicity and reduced survival, and conversely, GADD34 overexpression was protective in cells expressing mutant prion protein (PrPSc) [68]. Similarly, another study reported a detrimental effect of guanabenz in a mouse model of familial ALS, expressing mutant SOD1 [194]. Potential damage may appear in some tissues. For example, GADD34 inhibitors showed toxic effects in pancreatic β cells in both cellular and animal models [195,196].

Under chronical high levels of P-eIF2α, there is lengthy inhibition of protein synthesis, leading to depletion of essential short-lived proteins and extended upregulation of ATF4 with the consequent expression of pro-apoptotic factors. Therefore, an opposite approach of inhibiting the PERK pathway was also tried in several studies (reviewed in [30,102,103,197]). The highly effective PERK inhibitors GSK2606414 and GSK2656157 (Figure 2) were protective in studies of several neurodegenerative diseases. In a P301L tau mouse frontotemporal dementia model, PERK inhibition allowed recovery of protein synthesis and prevented neuronal loss, reducing behavioral symptoms [132]. The PERK inhibitors were also effective in mouse models of prion disease [130], PD [131], TBI [135] and AD [133] and in models of bone cancer and leukemia [198,199]. However, the GSK compounds were reported to also have off-target effects [134]. Another compound, echinacoside, was reported to be a PERK inhibitor as well and showed protection in a PD mouse model [138]. It also extended lifespan in a *Caenorhabditis elegans* AD model [136] and reduced accumulation of Aβ protein in *APPswe, PSEN1dE9* AD model mice expressing APP and PSEN1 mutations [137]. Consistently, conditional PERK knockout improved memory and synaptic plasticity in these AD model mice [51]. However, the outcome of PERK inhibition in AD is complicated by the fact that, similarly to ATF4, the translation of BACE1 is upregulated by phosphorylation of eIF2α. BACE1 proteolytically cleaves the amyloid precursor protein (APP) to produce Aβ. BACE1 levels are increased in AD mouse models and brains of AD patients [200].

Despite the positive influence of PERK inhibition in these studies, there were secondary side effects of pancreatic toxicity, because of the requirement of PERK activity to modulate ER stress which results from the high levels of insulin production in the pancreas [201,202]. A recent approach was to reduce PERK activity but only partially, by an inhibitory phosphorylation of its kinase activation loop. This was done using SC79, an activator of AKT, which is responsible for this phosphorylation [141]. Another approach was the development of compounds that inhibit the pathway downstream of P-eIF2α, partially restoring protein synthesis and inhibiting ATF4 translation. ISRIB was, thus, identified in a high throughput screen and turned out to bind eIF2B causing it to be resistant to P-eIF2α inhibition [203,204,205,206]. ISRIB showed protective effects in a model of prion disease [207], without pancreatic toxicity. ISRIB and an improved derivative, 2BAct, also had beneficial effects on a mouse model of the demyelinating VWMD. ISRIB stabilized and enhanced the remaining activity of mutant eIF2B in this disease [58,59]. ISRIB also increased survival in a cellular ALS model using mutant SOD1-expressing neurons, restoring general protein translation, but still allowing, to a degree, ATF4 translation. In the same study, GSK2606414 did not show the same benefit [142]. In an AD cellular model, ISRIB attenuated Aβ-induced neuronal cell death without affecting Aβ production [143]. Interestingly, ISRIB enhances memory, possibly by restoring protein synthesis, also improving working memory in old mice [208]. Long-term potentiation and cognition were restored and dendritic spines recovered by treatment with ISRIB or GSK2656157 of mice subjected to TBI [135,144]. Two repurposed drugs, trazodone and dibenzoylmethane (DBM), had better pharmacokinetic properties and a similar effect to ISRIB, showing neuroprotection in prion disease and in frontotemporal dementia mouse models and in a marmoset model of PD [145,146]. All these compounds act downstream of P-eIF2α, and therefore, counteract not only the effects of ER stress through PERK, but also the ISR through all four eIF2α kinases.

In the case of PERK pathway inhibition, not all studies showed beneficial effects. In a transgenic rat model of retinal degeneration, rats expressing P23H mutant rhodopsin, which causes ER stress and cell death, treatment with GSK2606414 enhanced photoreceptor apoptosis, even at low doses [209]. In the hAPP-J20 mouse model of AD ISRIB did not improve spatial learning and memory deficits [210].

## 6. Concluding Remarks

We can conclude that PERK pathway modulation using small molecule drugs is a very promising therapeutic approach for a wide variety of neurodegenerative diseases. It is surprising and puzzling that opposite approaches, both those that inhibit the pathway and those that activate it are reported to be beneficial for many diseases. We have tried to explain in this review the mechanisms that can explain these outcomes. The increase of eIF2α phosphorylation has been seen in virtually all neurodegenerative diseases where it was studied. In some, this could be the cause that activates apoptotic pathways. Therefore, PERK pathway inhibition in these cases can be beneficial. However, in other diseases, increased eIF2α phosphorylation can be a symptom of the cells fighting for their survival. It can actually be an insufficient cellular attempt to restore homeostasis by activating the initial adaptive phases of the PERK pathway. In these cases, PERK activation can be advantageous. Given the complexity of the pathway, different outcomes may result from subtleties in different disease models or in differences in the timing of drug delivery.

Approaches that strongly inhibit the pathway (PERK inhibitors) or that inhibit the turning off of the pathway (GADD34 inhibitors), although showing some initial benefit, can become toxic to many cell types. We believe that the most promising strategies for activation are those that boost the pathway without hindering its deactivation in the long term (PERK activators), and for inhibition, the most promising are those that partially inhibit the downstream effects of P-eIF2α.

As to whether it is best to activate or to inhibit, and for which diseases and conditions, this still remains an open question, pending forthcoming research.

## Figures and Tables

**Table 1:** UPR pathways in neurodegenerative diseases.

Neurodegenerative Disease	UPR Pathway	References
AD	Upregulation of the PERK pathway	[46,51]
	Activation of the ASK1 branch of the IRE1α pathway	[47,49,50]
PD	Increased levels of pPERK and P-eIF2α	[53,54,55]
	α-Synuclein aggregates were reported to interact directly with BiP and activate the UPR	[56]
	α-Synuclein binds RAB1, impairing COPII vesicular trafficking, thus inhibiting ATF6 activation	[57]
VWMD	Mutations in EIF2B (common branch of PERK pathway and ISR)	[58,59]
HD	Upregulation of PERK pathway	[60,61]
	Upregulation of IRE1 and ATF6 pathways	[61,62]
ALS	mSOD1 interacts with Derlin1 and activates ASK1 pathway	[63,64]
	mC9orf72 induces ISR	[65]
	mFUS induces ISR	[66]
	mTDP-43 increases ATF6 and XBP-1 activation	[67]
Prion disease	Mutant prion protein activates PERK pathway	[68]

**Table 2:** PERK pathway modulators.

Modulators (Compounds)	PERK Pathway Outcome	Neurodegenerative Diseases	Additional Targets
MK-28	Activation (via activating PERK)	HD [87]	
CCT020312	Activation (Nrf2 branch)	PSP [129]	
GSK260414	Inhibition (via inhibiting PERK)	Prion Disease [130], PD [131], FD [132], AD [133]	RIPK1 [134]
GSK2656157		Traumatic brain injury [135]	
Echinacoside (ECH)		AD [136,137], PD [138]	Ghrelin receptor [139], Androgen receptor [140]
SC79	Inhibition (activates AKT causing inhibitory phosphorylation of PERK kinase loop)	Prion Disease [141]	
2BAct	Inhibition (downstream of P-eIF2α, via eIF2B activation)	VWMD [58]	
ISRIB		ALS [142], VWMD [59], AD [143], TBI [144]	
Dibenzoylmethane (DBM)	Inhibition (downstream of P-eIF2α, similar activity to that of ISRIB)	FTD [145], Prion disease [145], PD [146]	Nrf2 [147], AMPK [148]
Trazodone HCl			T-type calcium channel [149], monoamine receptor [150]
Guanabenz	Activation (via inhibiting dephosphorylation of P-eIF2α, inhibits GADD34)	VWMD [117], ALS [116], PD [118,120]	ASICs [151]
PromISR-6 (guanabenz analog, target unknown)		HD [127]	Possibly activator of PERK and other eIF2α kinases [127]
Salubrinal		PD [53,56,114], TBI [113], HD [79]	CReP inhibitor, Dusp2 (PAC1) inhibitor [152]
Sephin1		ALS [123], Charcot-Marie-Tooth disease [123], MS [124], Prion Disease [125]	ASICs [151]
Raphin1	Activation (via inhibiting dephosphorylation of P-eIF2α, inhibits CReP)	HD [128]	
Bruceine D	Activation (Nrf2 branch, mechanism unknown)	PD [153]	Notch [154], JNK [155]
Naringenin (NAR)		PD [156], AD [157,158]	CRMP-2 [158]
Sulforaphane (SFN)	Activation (Nrf2 branch—acts on KEAP1, releasing Nrf2)	PD [159,160,161], HD [162], AD [163,164,165,166], MS [167], FRDA [168]	BACE1 [169], NF-κB [170]
Tertbuthyl-hydroquinene (tBHQ)	Activation (Nrf2 branch)	HD [171], AD [172], PD [173]	
Acetylenic tricyclic bis(cyanoenone) TBE-31	Activation (Nrf2 branch—binds KEAP1, thus releasing Nrf2)	FRDA [168]	
RTA-408 (omaveloxolone)		FRDA [174]	
Dimethylfumarate (DFM)		MS [175], PD [176,177]	MSK1 and RSK1 [178,179] and others [180]

## Data Availability

Not applicable.
